# Sex Modulates *Lactobacillus johnsonii* N6.2 and Phytophenol Effectiveness in Reducing High Fat Diet Induced mTOR Activation in Sprague-Dawley Rats

**DOI:** 10.3389/fmicb.2018.02649

**Published:** 2018-11-06

**Authors:** Danielle N. Kling, Evon M. DeBose-Scarlett, Leandro D. Teixeira, Salvador A. Gezan, Graciela L. Lorca, Claudio F. Gonzalez

**Affiliations:** ^1^Department of Microbiology and Cell Science, Genetics Institute, Institute of Food and Agricultural Sciences, University of Florida, Gainesville, FL, Unites States; ^2^School of Forest Resources and Conservation, Institute of Food and Agricultural Sciences, University of Florida, Gainesville, FL, Unites States

**Keywords:** high fat diet, *Lactobacillus johnsonii*, blueberry extract, mechanistic target of rapamycin, metabolic syndrome

## Abstract

Metabolic syndrome (MetS) is the underlying cause of some devastating diseases, including type 2 diabetes and cardiovascular disease. These diseases have been associated with over-activation of the mechanistic Target of Rapamycin (mTOR) pathway. This study utilizes a high fat diet (HFD) to induce MetS and to dissect the effects of a beneficial bacterium, *L. johnsonii* N6.2, and natural phenolics on mTOR complex 1 (mTORC1) expression compared to a reduced energy density diet (REDD). HFD significantly elevated MetS markers in males, as noted through an increase in weight, glucose levels, and triglyceride levels. Treatments were effective in reducing mTORC1-activating phosphorylation of pAKT-T308 and pAKT-S473 (*p* = 0.0012 and 0.0049, respectively) in HFD-fed females, with the combined treatments of *L. johnsonii* and phytophenols reducing phosphorylation below REDD-fed control levels, and significantly below HFD-fed control levels. Meanwhile, diet was the significant factor influencing male mTORC1-activating phosphorylation (*p* < 0.0001), as treatments were only effective in reducing phosphorylation in REDD-fed animals. Downstream analysis of mTORC1 activated genes phosphogluconate dehydrogenase (*pgd)* and phosphofructose kinase (*pfk)* followed this similar trend, enforcing the significant effect sex has on a treatments’ ability to modulate diet induced abnormalities. Analyzing mTORC1 stimulators such as insulin, inflammatory cytokines, and tryptophan, revealed no significant differences among groups. These results indicate that the effects observed on mTORC1 are a direct consequence of the treatments, and not exerted indirectly via the modulation of stimuli. This study highlights the potential use of commensal microorganisms and natural compounds in reducing the onset of metabolic diseases through mTORC1.

## Introduction

A poor diet high in fat, refined sugars, processed foods and “empty” calories has been defined as the hallmarks of a Western diet, which has been under increasing scrutiny for its negative impact on human health. Meanwhile, a Mediterranean diet, which emphasizes plant-based foods, is considered to be the healthy standard (Bach-Faig et al., 2011). The consumption of a Western diet has been closely linked with Metabolic Syndrome (MetS). MetS is characterized by five criteria, however, having three of the symptoms is required for a MetS diagnosis. These criteria are abdominal obesity, hypertriglyceridemia, low high-density lipoproteins, high blood pressure, and hyperglycemia (Lam and LeRoith, 2000). Having MetS puts individuals at a higher risk of other co-morbidities, such as type 2 diabetes and cardiovascular disease (Wilson, 2005). Abdominal obesity is arguably one of the most prevalent threats to human health, as obesity afflicts more than one-third of adults in the United States and is closely affiliated with MetS co-morbidities (Ogden et al., 2015). In fact, the occurrence of obesity, along with MetS co-morbidities, has nearly tripled since the 1970s (World Health Organization, 2005). It is predicted that if this trend continues, more than 50% of the United States population will be obese by 2030 (Finkelstein et al., 2012). Therefore, therapeutic strategies to control obesity and MetS are important in preventing the onset and reducing the prevalence of metabolic diseases.

Recent studies have determined that diet and its impact on the gut microbiota are closely related to obesity and metabolic diseases (Cani et al., 2008; Kim et al., 2012; Murphy et al., 2015). Therefore, bacteria that have been associated with beneficial characteristics could be used as therapeutic strategies in combating metabolic diseases. Studies focusing on the administration of beneficial microbes to diabetes-prone or MetS individuals have reported increased insulin sensitivity and reduced weight gain (Rajkumar et al., 2014; Stenman et al., 2014; Hulston et al., 2015). Administered bacteria have also been reported to decrease the inflammatory response, as evidenced through the reduction of inflammatory cytokines, which are associated with pre-disease onset. Our lab has been characterizing a strain of *Lactobacillus* since it was found to be negatively correlated with type 1 diabetes development when comparing the intestinal microbiota of Bio-Breeding diabetes prone to diabetes resistant rats (Roesch et al., 2010). The ability of *L. johnsonii* to decrease inflammation, modulate the tryptophan catabolism pathway, and release phytophenols from dietary fiber could aid in modulating host regulatory pathways (Kin et al., 2009; Valladares et al., 2010, 2013). Phytophenols are plant-derived molecules, some of which have been described to inhibit mechanistic Target of Rapamycin (mTOR) pathway functions (Castillo-Pichardo and Dharmawardhane, 2012; Kresty et al., 2015; Park et al., 2016). Phytophenols, along with inflammatory cytokines, and amino acids such as tryptophan all have been described as having the ability to affect mTOR pathway activity.

mTOR is a serine/threonine kinase in the PI3K/AKT pathway that responds to growth factors, ATP, cytokines, amino acids and oxygen levels. This enzyme presents itself in two multi-protein complexes, mTORC1 and mTORC2, that collectively modulate functions that enable a cell to proliferate, grow, and survive, while repressing autophagy. A common path to mTORC1 activation requires the activation of PI3K, which, through multiple interactions, leads to phosphorylation of AKT at Thr-308 and its partial activation (Alessi et al., 1997). Full activation of AKT requires subsequent phosphorylation at Ser-473 by mTORC2 (Sarbassov et al., 2005). Active AKT phosphorylates and subsequently inhibits the tuberous sclerosis complex (TSC), allowing Rheb to activate mTORC1 (Potter et al., 2002; Inoki et al., 2003). Activation of mTORC1 leads to an increase in protein synthesis, lipid biosynthesis, and a decrease in autophagy primarily through two main effectors: p70S6 kinase 1 (S6K) and eIF4E binding protein (4EBP). The phosphorylation of these effectors promote translation initiation at 5’cap mRNAs (Gingras et al., 1999; Holz et al., 2005). The activation of mTORC2 controls cytoskeletal organization, glucose metabolism, and apoptosis through several key effectors. Besides AKT, mTORC2 is known to phosphorylate PKC, a cytoskeleton regulator, and SGK1, a regulator of ion transport and cell survival (Jacinto et al., 2004; García-Martínez and Alessi, 2008). However, its phosphorylation of AKT is arguably mTORC2’s most important role, as this unlocks AKT’s ability to inhibit FoxO1/3a transcription factors, the metabolic regulator GS3Kβ, and the mTORC1 inhibitor TSC. Balance of this pathway is essential, as deregulation has been heavily implicated in common pathological conditions, such as cancer, type 2 diabetes, and nonalcoholic fatty liver disease (NAFLD) (Saxton and Sabatini, 2017). This is important when considering that these conditions are among the top leading causes of death in the United States (Health United States, 2016; With Chartbook on Long-term Trends in Health, 2017). Therefore, modulation of this pathway could be critical in treating or preventing several conditions whose chronic complications are important modern societal burdens.

The overall goal of this study is to evaluate the role of a beneficial bacterium with natural food ingredients and dissect its effects on mTORC1 activation. This study uses a high fat diet (HFD) to promote MetS and evaluate its effects on mTOR pathway activation compared to a reduced energy density diet (REDD). Phytophenols are administered to animals through blueberry extracts, which contains one of the highest phytophenol contents of all edible plants and has a great diversity of phytophenols. The administration of phytophenols and *L. johnsonii* N6.2 was evaluated individually, as well as in combination on its ability to modulate mTORC1-activating phosphorylations and downstream gene expression. To determine if differences in the pathway expression were due to external stimuli or treatment, some common mTORC1 stimulating signals were evaluated.

## Materials and Methods

### Cell Culture

*L. johnsonii* N6.2 was grown in MRS medium (Remel, Lenexa, KS, United States) as previously described (Valladares et al., 2013). After incubation at 37°C, cells were pelleted by centrifugation, washed twice with PBS, resuspended in PBS, aliquoted and stored at -80°C for feeding assays. Colony forming units (CFU)/mL for aliquots were determined by randomly selecting three aliquots and performing serial plate dilutions with PBS on MRS agar. The average CFU/mL of the three aliquots was used as the concentration.

### Blueberry Extraction and Phenol Enrichment

Freeze-dried Tifblue/Rubel 50%/50% *w/w* blend of blueberries was used as a phytophenol extraction source, protocol from Grace et al. (2014). Briefly, blueberries were blended (Waring, Inc., Torrington, CT, United States) at a ratio of 1:12 blueberry powder to acidified 70% methanol (0.5% acetic acid) *w/v* for 2 min. The mixture was centrifuged for 20 min at 4000 rpm and the supernatant was transferred to a round-bottom flask. The extraction of the pellet was repeated 2 more times and the extracts (i.e., supernatant) were combined. The extract was neutralized, rotary evaporated (Buchi Rotavapor, New Castle, DE, United States), frozen, then lyophilized (Labconco, Kansas City, MO, United States). Blueberry extract powder was resuspended in PBS, aliquoted into black microcentrifuge tubes (Argos Technologies, Vernon Hills, IL, United States), and stored at -80°C until use.

### Animal Models and Housing Conditions

Animal models used in this study were Sprague-Dawley weaned pups (Envigo, East Millstone, NJ, United States). All animals were housed on ALPHA-dri bedding, under identical conditions with 12 h light-dark cycles and received water and food (specially formulated HFD or REDD, see Feeding Design) *ad libitum*. Animal housing standards were maintained as prescribed by the Association for Assessment and Accreditation of Laboratory Animal Care. Animal protocols were approved by the University of Florida Institutional Animal Care and Use Committee.

### Animal Feeding Design

Pregnant females were obtained from Envigo. Females gave birth, and pups and their mother were housed in the same cage until weaning. After 21 days, pups from each litter were randomly divided into eight treatment groups: HFD + vehicle control, REDD + vehicle control, REDD + blueberry extract (BBE), REDD + *L. johnsonii* (Ljo), REDD + BBE + Ljo, HFD + BBE, HFD + Ljo, HFD + BBE + Ljo. Diets were formulated with the help of a nutritionist at Envigo following the Nutrient Requirements of Laboratory Animals (National Research Council (US) Subcommittee on Laboratory Animal Nutrition, 1995). Reduced energy density diet (TD.150312) and HFD (TD.150313) had the minimally acceptable antioxidant (i.e., vitamins) supplementation as not to interfere with downstream analyses. Immediately after weaning, treatments were administered three times a week. *L. johnsonii* N6.2 was administered at a concentration of 10^8^ CFU/d suspended in 100 μL PBS, meanwhile blueberry extract phenols were administered 25 mg/kg body weight suspended in 100 μL PBS. Blood glucose levels were monitored using a TRUEresult blood glucose monitor (Nipro Diagnostics, Fort Lauderdale, FL, United States). Blood triglycerides levels were monitored using a CardioChek analyzer (PTS Diagnostics, Indianapolis, IN, United States). Rats were weighed weekly. Assay was conducted for 15 weeks at which point rats were sacrificed by CO_2_ inhalation followed by immediate decapitation. Blood was collected, allowed to coagulated at room temperature for 1 h, and centrifuged at 4°C for 10 min at 2000 × *g*. Supernatant (serum) was aliquoted and flash-frozen in liquid nitrogen. Tissues were excised and rinsed with ice-cold PBS. In order to preserve tissues, they were flash-frozen in liquid nitrogen or preserved in RNA*later* (Thermo Fisher Scientific, Waltham, MA, United States) for mRNA analysis. All Samples were stored at -80°C until use.

### Determination of Total Phenolics

Total phenolic content of blueberry extract aliquots were quantified using the Folin-Ciocalteau’s reagent (Singleton and Rossi, 1965). Briefly, sample or standards (15 μL) were diluted in 240 μL of dH_2_O in a 96-well microplate. Then, 15 μL of 2N Folin-Ciocalteau’s reagent (Sigma-Aldrich, St. Louis, MO, United States) was added and the mixture was incubated for 5 min at room temperature. The distinctive blue color was developed by adding 30 μL of 15% Na_2_CO_3_ solution. The plate was incubated in the dark for 2 h at room temperature before measuring the absorbance at 725 nm using a Sinergy HT microplate reader (Biotek, Winooski, VT, United States). The total phenolic concentration was determined from a standard curve using gallic acid. The polyphenol concentration is expressed at micrograms of gallic acid equivalent per mL of sample.

### Quantitative Real Time PCR Analysis

Stomach or liver tissue preserved in RNAlater was used for RNA extraction using the RNAqueous Phenol-Free Total RNA Isolation kit (Thermo Fisher Scientific, Waltham, MA, United States). Briefly, 20 mg of tissue was homogenized using a tissue homogenizer (Omni International, Kennesaw, GA, United States) in the kit’s Lysis/Binding solution, and the rest was performed per the manufacturer’s instructions. Contaminating DNA from the samples was eliminated via DNase Max kit (Qiagen, Valencia, CA, United States). Remaining DNA contamination was tested by qPCR analysis of the presence of housekeeping genes: β-actin, glyceraldehyde 3-phosphate dehydrogenase (Gapdh), and ribosomal protein lateral stalk subunit P0 (Rplp0). If no subsequent DNase treatments were needed, cDNA was synthesized using iScript cDNA Synthesis Kit (Bio-Rad, Hercules, CA, United States). The qRT-PCR assays were performed using PowerUp SYBR Green Master Mix (Applied Biosystems, Foster City, CA, United States) in a QuantStudio6 machine (Applied Biosystems, Foster City, CA, United States). The changes in gene expression (*C*_t_ values) between treatments and HFD controls were compared to REDD controls using the 2^-ΔΔ*C_t_*^ method. Amplification of *rplp0* was used as an internal control. The primers used during the qRT-PCR experiments are described in Table 1. Primers were designed in this study using NCBI Primer-BLAST, unless otherwise indicated.

**Table 1:** Primers utilized in qRT-PCR experiments.

Gene	Forward sequence (5′ → 3′)	Reverse sequence (5′ → 3′)	Source
*gapdh*	ATGACTCTACCCACGGCAAG	GGAAGATGGTGATGGGTTTC	This work
*β-actin*	CGGCAATGAGCGGTTC	AGCACTGTGTTGGCATAGAGG	This work
*rplp0*	CATCTCCCCCTTCTCCTTCGG	CCCTCTAGGAAGCGAGTGTG	This work
*pfk*	CCGTATCCCCAAGCAACAGT	CAGCTTGCCTACGTCTGACA	This work
*pgd*	GATCATGGGTTTGTGGTCTGTG	TCCTGGCATCTTCTTGTCGT	This work
*srebp1c*	ACGACGCAGCCATGGATTG	CCAGCATAGGGGGCATCAAA	This work
*ceacam1*	TAGCAGCGCTGGCATACTTC	CCAGATTGTGGCTGGAGGTT	Wang et al., 2016
*tnfa*	GCCGATTTGCCATTTCATAC	TGGAAGACTCCTCCCAGGTA	Lee et al., 2017
*il1b*	TCCTCTGTGACTCGTGGGAT	TCAGACAGCACGAGGCATTT	Xie et al., 2016
*il6*	CCCAACTTCCAATGCTCTCCT	GGATGGTCTTGGTCCTTAGCC	This work


### Western Blot Analysis

Total protein extracts were prepared from tissue samples using Radio Immunoprecipitation Assay Buffer containing 150 mM NaCl, 50 mM Tris (pH 8), 1% Triton X-100, and 0.1% sodium dodecyl sulfate (SDS), and Halt Protease and Phosphatase Inhibitor Cocktail (Thermo Fisher Scientific, Waltham, MA, United States). After tissue homogenization, lysates were allowed to shake at 4°C for 2 h and then centrifuged at 12,000 × *g* for 10 min at 4°C. Supernatant was transferred to a new tube and the protein concentration was quantified with the Bio-Rad protein assay, using bovine serum albumin (BSA) as a standard (Bio-Rad). Thirty microgram of proteins were separated using 12.5% *v/v* sodium dodecyl sulfate-polyacrylamide gel electrophoresis (SDS-PAGE) and transferred to a polyvinylidene difluoride (PVDF) membrane (Bio-Rad) using a semi-dry blotting unit (Fisher Scientific, Hampton, NH, United States). Primary antibodies AKT, pAKT-T308, pAKT-S473, p70S6K, pS6K-T389, and GAPDH were purchased from Cell Signaling Technology (Danvers, MA, United States), while the horseradish peroxidase-conjugated secondary and β-actin antibody were purchased from Abcam (Cambridge, MA, United States). Antibodies were used according to the manufacturer’s instructions with slight modifications. Briefly, membranes probing for unphosphorylated markers were blocked in 5% non-fat dry milk, while membranes probing for phosphorylated markers were blocked in 5% BSA (Gold Biotechnology, St. Louis, MO, United States) for 1 h at 4°C. Membranes were washed three times in 0.1% Tween-20 (Sigma-Aldrich, St. Louis, MO, United States) Tris buffered saline (TBS-T). Primary antibody incubation was done overnight at 4°C in the same blocking solutions and with a primary antibody dilution of 1:25,000. Membranes were washed three times with TBS-T and incubated in 5% non-fat dry milk with a secondary antibody dilution of 1:25,000 for 1 h at 4°C. ProSignal Femto solution (Genesee Scientific, San Diego, CA, United States) was used for visualization by the automatic imager FluorChem R (ProteinSimple, San Jose, CA, United States). The relative intensity of the bands visualized on the membranes was quantified using ImageJ software (Free Java software provided by the National Institutes of Health, Bethesda, MD, United States). GAPDH or β-actin was used to normalize the bands of each sample.

### Histology and Microscopy

Hematoxylin and eosin (H&E) stained liver slides of male and female HFD and REDD rats were examined microscopically (×400 magnification) for fat deposits. Samples were first fixed in 10% neutral buffered formalin solution (Sigma-Aldrich, St. Louis, MO, United States) overnight and then placed in 70% ethanol solution until paraffin embedding. The embedding process was performed by the University of Florida Molecular Pathology Core using automatic processors (VIP6, Leica Biosystems Inc., Buffalo Grove, IL, United States) with graded ethanol (70–100%) and xylene. Samples were sectioned at 4 μm thickness, fixed to a slide, and H&E stained.

### Serum Analysis

Serum was preserved as described in Animal Feeding Design. Quantification of tryptophan was performed using global high-performance liquid chromatography and mass spectrometry (LC-HRMS/HRMS) at the Southeast Center for Integrated Metabolomics at the University of Florida. Rat Insulin ELISA kit (Mercodia, Winston Salem, NC, United States) and Rat Adiponectin ELISA kit (Abcam) were performed according to manufacturer’s instructions.

### Statistical Analysis

For the variables *srebp1c, ceacam1* and adiponectin linear mixed models that included the fixed term of diet and random term for cage were fitted for males and females, separately. In the case of pAKT-T308, pAKT-S473, pS6k, *pgd, pfk*, tryptophan, and insulin, the previous model was extended to include the fixed factors of treatment, and its interaction. The variables insulin, *pfk* and *pgd* were log-transformed in order to approximate normality of residuals. To evaluate statistical differences between males and females, an additional model was fitted for the variables *srebp1c, ceacam1*, pAKT-T308, pAKT-S473, pS6K, *pgd*, and *pfk*. This model included the same factors as above, with the addition of sex and all their two- and three-way interactions. Here, different error variances were assumed for each of the levels of sex. A repeated measures analysis was performed over the weekly monitoring of weight, glucose, and triglycerides for each subject. The linear mixed model included the fixed factors of week, diet and its interaction, and a random factor of cage. Residuals were modeled with an autoregressive error structure of order 1 for each of the subjects. Significance of model terms and treatment comparisons were done with a significance level of α = 0.05, and all models were fitted using SAS v. 9.4 with the procedure MIXED and degrees of freedom were adjusted using the Kenward–Rogers correction. *Post hoc* multiple comparisons were performed with least significant differences. Normality and homogeneity of residuals was assessed by exploring residual plots. GraphPad Prism 5.01 Software (GraphPad Software, La Jolla, CA, United States) was used for data visualization.

## Results

**FIGURE 1:**
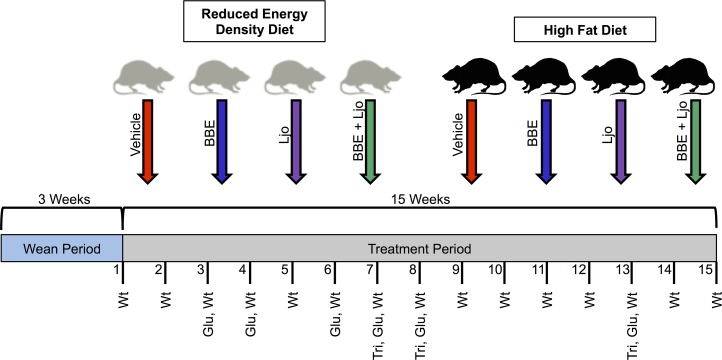
Feeding assay design. Pregnant females gave birth, and pups were weaned after 3 weeks and separated into two diet groups (reduced energy density diet and high fat diet), each with four treatments (vehicle control, BBE = blueberry extract*;* Ljo = *L. johnsonii*; BBE + Ljo = blueberry extract + *L. johnsonii*). Animals were treated three times a week for 15 weeks, during which regular monitoring of weight (wt), glucose levels (glu), and triglycerides levels (tri) were assessed.

Sprague-Dawley rats are valuable models for diet-based studies. A HFD able to induce MetS for these animal models usually consist of 40–60% energy derived from fat, based on various studies and rodent diet distributors. Comparatively, 5–15% of energy comes from fat in a standard rodent diet. The macronutrient breakdown and energy density for the diets utilized in this study are described in Table 2. To investigate the effects of diet on MetS and host regulatory pathways, a feeding assay was designed using two specially formulated diets supplemented with treatments of *L. johnsonii* and blueberry phytophenols (Figure 1).

**Table 2:** Macronutrient breakdown and energy densities for reduced energy density diet (REDD) and high fat diet (HFD).

	Reduced energy density diet (REDD)	High fat diet (HFD)
% Kcal from protein	26.3	13.6
% Kcal from carbohydrates	56.2	28.3
% Kcal from fat	17.5	58
Kcal/g	2.7	5.1


### High Fat Diet Increased MetS Markers Among Males

**FIGURE 2:**
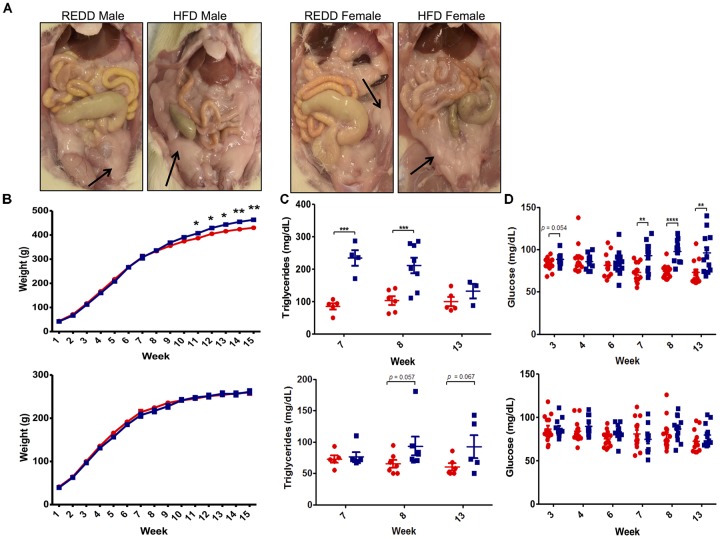
The effects of formulated reduced energy density diet (REDD) and high fat diet (HFD) on MetS markers. **(A)** Representative images of the abdominal cavity highlighting the excess adipose tissue found in HFD fed animals compared to REDD fed animals. Arrows identify adipose tissue. **(B)** Weight in grams in male (top) and female (bottom) fed a REDD and HFD throughout the 15 weeks of the assay. *n* = 12–14. **(C)** Blood triglyceride levels for male (top) and female (bottom). **(D)** Blood glucose levels for male (top) and female (bottom). Red circle, reduced energy density diet; blue square, high fat diet. Numerical data are summarized as means ± SEM. ^∗^*p* < 0.05, ^∗∗^*p* < 0.01, ^∗∗∗^*p* < 0.001, ^∗∗∗∗^*p* < 0.0001.

Since a HFD is a major contributing factor to MetS, symptoms associated with this disorder were evaluated. Diagnosis of MetS requires the presence of any of the three following criteria: abdominal obesity, hyperglycemia, low high-density lipoprotein, high blood pressure, and hypertriglyceridemia (Lam and LeRoith, 2000). Upon sacrifice, excessive visceral fat was noted in HFD-fed animals, particularly among males (Figure 2A). This correlated well with observed weight, however, only HFD-fed males weighed significantly more than REDD-fed males, and no significant difference was detected between the two diet female groups by the end of the assay (Figure 2B). Beginning at week 11, differences in weight between HFD-fed males and REDD-fed males became statistically significant, continuing throughout the length of the study. Interestingly, this trend continued when evaluating triglyceride and glucose levels throughout the study (Figures 2C,D). Even more interesting was the observation that significant differences in triglyceride levels precede the significant differences in weight gain and glucose levels among the diets in males. Even though most weeks HFD-fed females experienced, on average, higher triglyceride and glucose levels than REDD-fed females, at no time were there significant differences detected among triglyceride levels between the diets. Therefore, the results support that the HFD utilized in this study was successful in increasing the MetS factors of weight, glucose levels, and triglyceride levels, most notably among males.

### Morphology of Affected Organs Is Altered in a HFD

**FIGURE 3:**
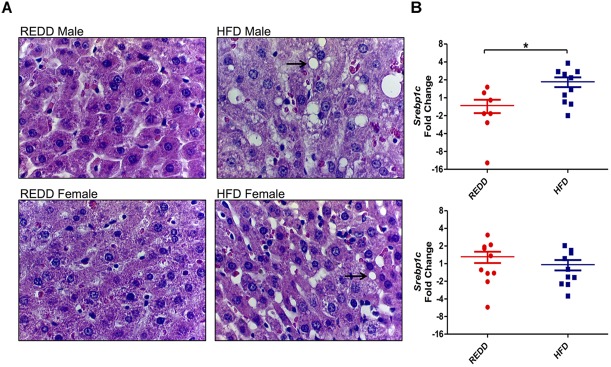
HFD induces morphological changes in the liver. **(A)** Representative liver histology sections at 400× magnification of REDD-fed male (top left), HFD-fed male (top right), REDD-fed female (bottom left), and HFD-fed female (bottom right) rats. Arrows identify fat deposits. **(B)** Hepatic *Srebp1c* gene expression quantified by qRT-PCR between male (top) and female (bottom). *Rplp0* expression was used as an internal standard. Results are expressed as Log_2_(Fold Induction) with repression values (i.e., <1) expressed as its negative reciprocal (i.e., 0.5 = -2). Numerical data are summarized as means ± SEM. ^∗^*p* < 0.05. REDD = reduced energy density diet; HFD = high fat diet.

Based on the differences observed in MetS markers, the ability of the HFD to affect the physiology of susceptible organs was evaluated. A HFD, obesity, and MetS are connected to other co-morbidities, one being the increased deposition of fat in the liver, which could lead to NAFLD (Hsiao et al., 2007). Therefore, the ability of the HFD utilized in this study to increase fat deposits and alter the metabolic functions of enzymes in the liver was evaluated. While both males and females experienced more liver fat deposits when fed a HFD, males appear to be more susceptible to the effects of a HFD compared to females (Figure 3A). Fat deposits in males are bigger and more numerous than females. These results reflect the MetS data obtained. These observations were positively correlated with the gene expression analysis of sterol regulatory element binding protein (SREBP1c*)*. SREBP1c is a transcription factor that directly promotes *de novo* lipogenesis by increasing the expression of lipogenic genes, such as acetyl-CoA carboxylase, stearoyl-CoA desaturase 1, and fatty acid synthase (Mauvoisin et al., 2007). Studies have highlighted the importance of *de novo* lipogenesis in the accumulation of hepatic triglycerides, especially those with NAFLD (Donnelly et al., 2005). Males fed a HFD experienced a 2.5-fold increase in *srebp1c* expression levels than those fed a REDD, while HFD-fed females did not differ from the *srebp1c* expression levels in REDD-fed animals (Figure 3B). When analyzing the sexes together a HFD is able to considerably increase *srebp1c* levels compared to REDD controls (*p* = 0.057). These results are in agreement with the observation of significant hepatic lipid accumulation in males, and mild accumulation in females.

### HFD Promotes Early Stages of Disease Progression

**FIGURE 4:**
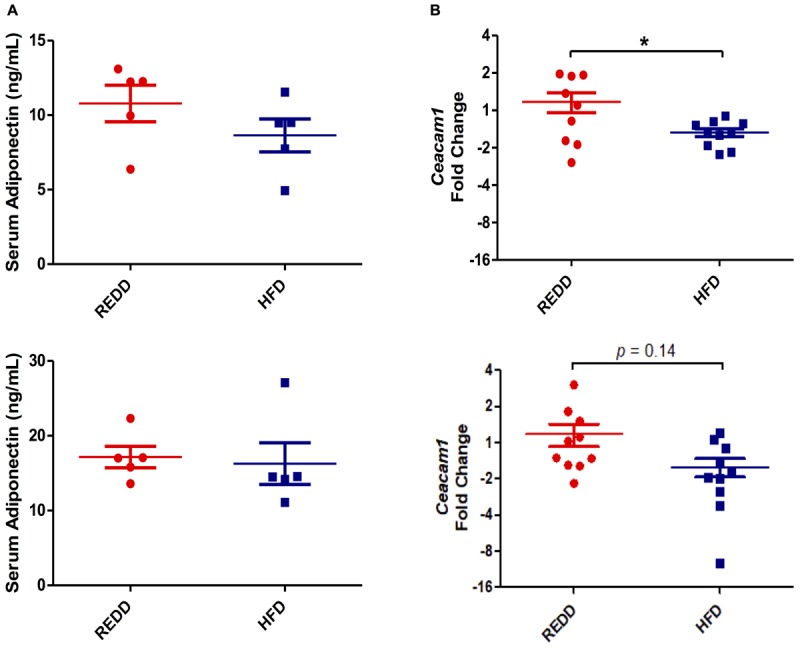
HFD alters insulin signaling. **(A)** Quantification of serum adiponectin levels via ELISA for male (top) and female (bottom). **(B)** Hepatic *Ceacam1* gene expression quantified by qRT-PCR for male (top) and female (bottom). *Rplp0* expression was used as an internal standard. Results are expressed as Log_2_(Fold Induction) with repression values (i.e., <1) expressed as its negative reciprocal (i.e., 0.5 = -2). Numerical data are summarized as means ± SEM. ^∗^*p* < 0.05. REDD, reduced energy density diet; HFD, high fat diet.

In order to define the disease state of animals, the inflammatory status of the liver was evaluated by evaluating TNF-α levels. Although HFD-fed animals experienced increased fat deposits in the liver, gene expression analysis of TNF-α, an inflammatory cytokine heavily associated with NASH, did not reach significant levels for quantification in the liver indicating that animals are still in the beginning stages of steatosis and have not progressed into NASH (Hui et al., 2004). Investigation into circulating adiponectin levels, an adipose hormone involved in lipid oxidation and glucose metabolism, revealed that levels did not differ between diets among females and males (Figure 4A). A reduction in adiponectin is highly associated with insulin resistance, obesity, and MetS making this marker an important one to follow in metabolic diseases. Though no differences were found between diets among male and females, a significant difference between sexes was observed in HFD-fed animals (*p* = 0.018). Therefore, the results obtained suggest that animals fed a HFD may be in the early stages of disease progression, and males have progressed more than females.

The HFD diet utilized was evaluated on whether it was able to interfere with insulin signaling. Carcino-Embryonic Antigen-related cell Adhesion Molecule 1 (CEACAM1) regulates insulin sensitivity by promoting hepatic insulin clearance. Our results of hepatic *ceacam1* expression levels showed decreased levels in animals fed a HFD, regardless of sex (Figure 4B). However, the reduction in *ceacam1* was only significant among males, which experienced a 57% decrease compared to REDD-fed animals. As observed with *srebp1c* levels, there was a significant difference between HFD-fed animals and REDD-fed animals (*p* = 0.0039), regardless of sex. This data suggests that insulin sensitivity may be compromised even at early stages of disease progression in HFD-fed animals.

### HFD Activates mTOR and Is Mitigated by Dietary Supplements

**FIGURE 5:**
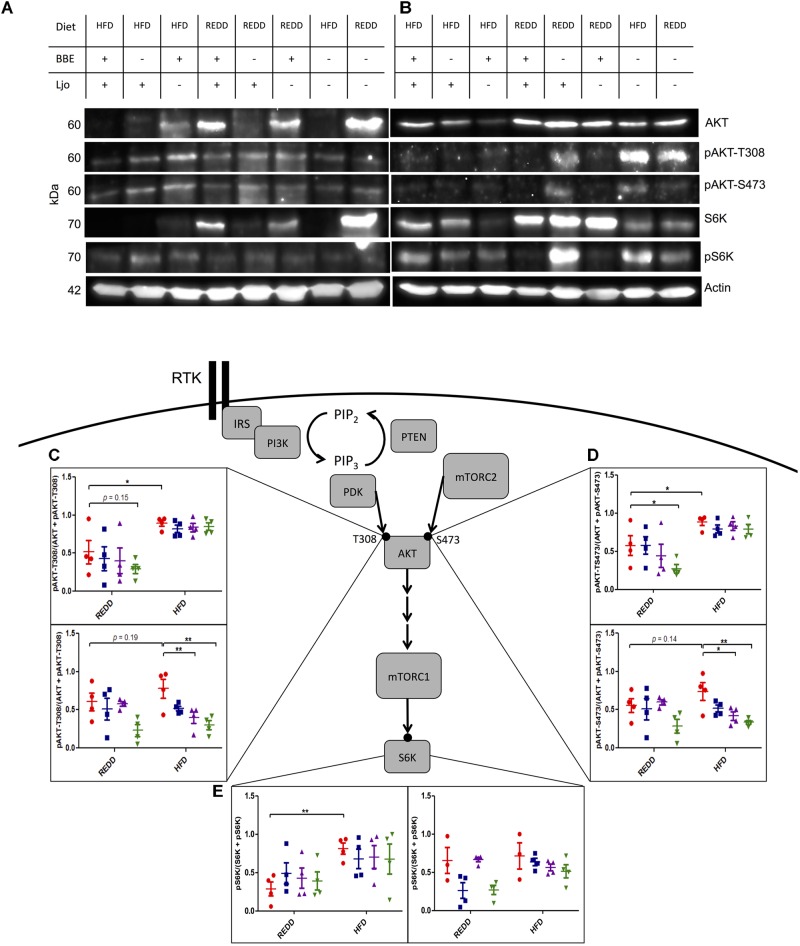
Treatments effect mTORC1 activation in the stomach. Representative Western blot of the pattern observed in REDD and HFD treated **(A)** males and **(B)** females comparing the expression of the mTORC1 pathway in the stomach. Actin was used as an internal control. All samples were run in parallel. Images were minimally adjusted in contrast and brightness. Relative quantification of **(C)** pAKT-T308 for male (top) and female (bottom), **(D)** pAKT-S473 for male (top) and female (bottom), and **(E)** pS6K for male (left) and female (right) by densitometry analysis of Western blots. Proteins were extracted by homogenizing tissue in RIPA buffer. A Bradford curve using BSA as a standard was used for protein quantification and 30 μg of protein was separated on a 12.5% SDS-PAGE gel. Actin was used an as internal standard to ensure consistent loading and to normalize all samples. ImageJ was used to quantify intensity of bands. Phosphorylation levels were calculated by dividing the phosphorylation by the unphosphorylated and the phosphorylated form of the protein. Numerical data are summarized as means ± SEM. ^∗^*p* < 0.05, ^∗∗^*p* < 0.01. Red circle, control; blue square, blueberry extract; purple triangle, *L. johnsonii*; green triangle, blueberry extract + *L. johnsonii*. REDD, reduced energy density diet; HFD, high fat diet; BBE, blueberry extract; Ljo, *L. johnsonii*.

Since mTORC1 has been heavily implicated in metabolic and proliferative diseases, it was our goal to determine the ability of a HFD, supplemented with a beneficial symbiont and phytophenols, to modulate mTORC1 activity. Since the consumption of a HFD is associated with inflammation, insulin resistance, and increased nutrient intake, it was hypothesized that HFD-feeding can be a stimulator of mTORC1 expression. Indeed, it has been reported that a HFD can increase mTORC1 activation (Khamzina et al., 2005). It is known that mTORC1 is especially active in liver, pancreatic, and muscle tissues (Laplante and Sabatini, 2012). However, since this pathway is expressed in nearly every organ, we expanded our examination of mTORC1 pathway to include stomach, jejunum, ileum, colon, cecum, pancreas, liver, and spleen in order not to oversee notable differences in expression levels among other tissues. Evaluating differences in mTORC1 biomarker abundance via Western blot in REDD-fed (Supplementary Figure 1) and HFD-fed (Supplementary Figure 2) males did not indicate substantial differences between these two diets among any tissue. However, comparing REDD-fed (Supplementary Figure 3) and HFD-fed (Supplementary Figure 4) females, multiple tissues indicated higher expression of the mTORC1 pathway, including liver, colon, cecum and pancreas (Supplementary Figure 5). Further investigation revealed an interesting expression pattern between male (Figure 5A) and female (Figure 5B) stomach tissue lysates. The analysis of stomach tissue revealed an increase in activating mTORC1 phosphorylations among HFD-fed animals, regardless of sex (Figures 5C–E). This difference was reflected down the mTORC1 pathway, as upper pathway phosphorylations of AKT at T308 (*p* < 0.0001) and at S473 (*p* < 0.0001), and lower pathway phosphorylation of S6K (*p* = 0.006) showed higher expression in HFD animals.

Diets were supplemented with *L. johnsonii* and/or blueberry extract to determine their effects on mTORC1 activity. Blueberry extract consists of enriched phenolic fractions, of which 77% of phenolics were retained from the blueberry freeze-dried powder. The administration of treatments was successful in reducing pAKT-T308 (*p* = 0.0012) and pAKT-S473 (*p* = 0.0049) in HFD females, but not males. In the case of males, diet was the overarching factor mediating phosphorylation on AKT-T308 (*p* < 0.0001), AKT-S473 (*p* < 0.0001) and downstream S6K (*p* = 0.0024). Therefore, treatments were able to overcome the effects exerted by a HFD in females, but a HFD trumped the potential effects of treatments in males. Analysis of Western blot bands also revealed that blueberry extracts or *L. johnsonii* supplementation alone to a HFD was successful in reducing phosphorylation on AKT down to REDD-fed levels in females (Figures 5C,D). However, a significant reduction was observed when these treatments were administered together when compared to HFD-fed control females. Even the HFD with the combined treatments showed, on average, lower AKT phosphorylation levels when compared REDD-fed control females. When observing downstream mTORC1 activity through the phosphorylation of S6K in females, we found that the pattern of phosphorylation reflects those of AKT, although treatments were not able to reduce phosphorylations down to the same levels (Figure 5E). Individual treatments were able to slightly reduce the phosphorylation of S6K, and the combined treatment was able to reduce it down to REDD control levels among females. Although treatments were unable to reduce mTORC1 phosphorylations among HFD-fed males, treatments reflected the same pattern in REDD-fed males as HFD-fed females. Albeit mildly, blueberry extract and *L. johnsonii* were able to individually reduce AKT phosphorylations, but only the combined treatment reduced them down to below REDD-control levels. This could lead to lower levels of mTORC1 activation and downstream gene expression levels.

**FIGURE 6:**
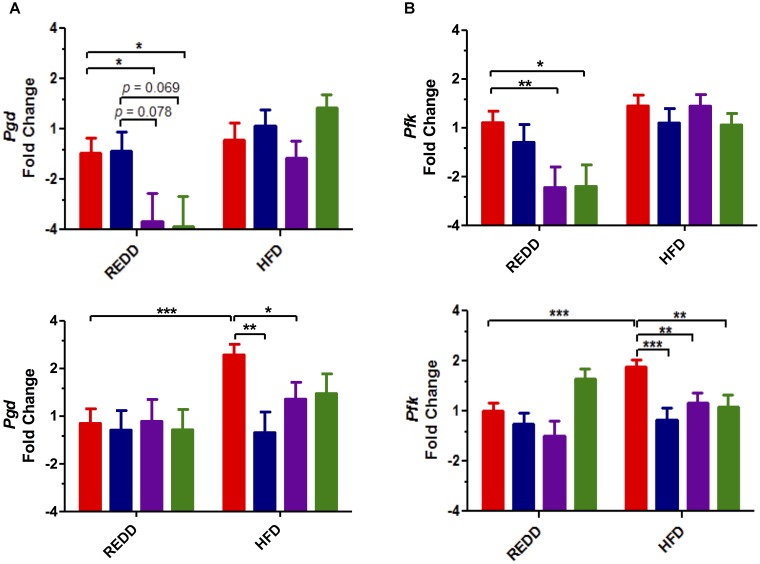
Treatments affect the transcription of mTORC1-activated genes in the stomach. **(A)** *Pgd* gene **(B)** *Pfk* gene expression quantified by qRT-PCR in male (top) and female (bottom). *Rplp0* was used as an internal standard. Results are expressed as Log_2_(Fold Induction) with repression values (i.e., <1) expressed as its negative reciprocal (i.e., 0.5 = -2). The number of animals analyzed for REDD Control, REDD + BBE, REDD + Ljo, REDD + BBE + Ljo, HFD Control, HFD + BB, HFD + Ljo, HFD + BBE + Ljo are as follows: *Pgd* Male, *n* = 9, 5, 8, 7, 5, 5, 7, 7, respectively, *Pgd* Female, *n* = 9, 6, 4, 5, 9, 5, 5, 3, respectively, *Pfk* Male, *n* = 10, 5, 8, 7, 9, 6, 8, 10, respectively, *Pfk* Female, *n* = 10, 6, 4, 4, 9, 5, 5, 4, respectively. Numerical data are summarized as means ± SEM. ^∗^*p* < 0.05, ^∗∗^*p* < 0.01, ^∗∗∗^*p* < 0.001. Red bar, control; blue bar, blueberry extract; purple bar, *L. johnsonii*; green bar, blueberry extract + *L. johnsonii*. REDD, reduced energy density diet; HFD, high fat diet.

Even more downstream of mTORC1, activation of this complex is responsible for inducing the transcription of genes in metabolic pathways that induce cell growth. Therefore, to uncover the biological impact of mTORC1 pathway phosphorylations, the expression of affected genes was determined. Two pathways in which mTORC1-mediated activation have been shown to increase gene expression are glycolysis and the pentose phosphate pathway (Düvel et al., 2010). Evaluation of phosphofructokinase (*pfk*), from the glycolysis pathway, and phosphogluconate dehydrogenase (*pgd*), from the oxidative arm of the pentose phosphate pathway, showed increased expression of these genes in HFD-fed controls compared to REDD-fed controls, most notably in females (Figures 6A,B). HFD-fed females experienced a 1.9- and 1.6-fold increase in *pgd* and *pfk* levels, respectively, compared to REDD-fed animals. Similar to what was observed with the phosphorylation patterns, treatments were able to reduce the expression of *pgd* and *pfk* among HFD-fed females. Meanwhile, diet (*pgd, p* = 0.0023; and *pfk, p* = 0.0004) seems to have a greater effect on male rats than treatments, as treatments were not as successful in reducing the expression of these genes in a HFD context. However, the effect of *L. johnsonii* and the combined treatments of blueberry extract and *L. johnsonii* were able to significantly reduce the expression of these genes in REDD-fed male rats. In agreement with the Western blot analysis, treatments utilized in this study were successful in reducing HFD-induced mTORC1 gene expression among females and REDD-fed males. Activation of mTORC1 has been known to promote protein synthesis, lipid synthesis and decrease autophagy, functions a cell needs to grow and proliferate. These functions require significant amounts of energy and resources, therefore increasing the gene products for metabolic pathways that produce ATP, NADH, NADPH, and precursors to nucleic acids and amino acids are essential for growth. Treatments were successful in reducing mTORC1-activating pathway phosphorylations and reducing downstream gene expression, thereby decreasing the growth and proliferative nature of mTORC1 activation.

### Evaluation of mTORC1 Inducers

**FIGURE 7:**
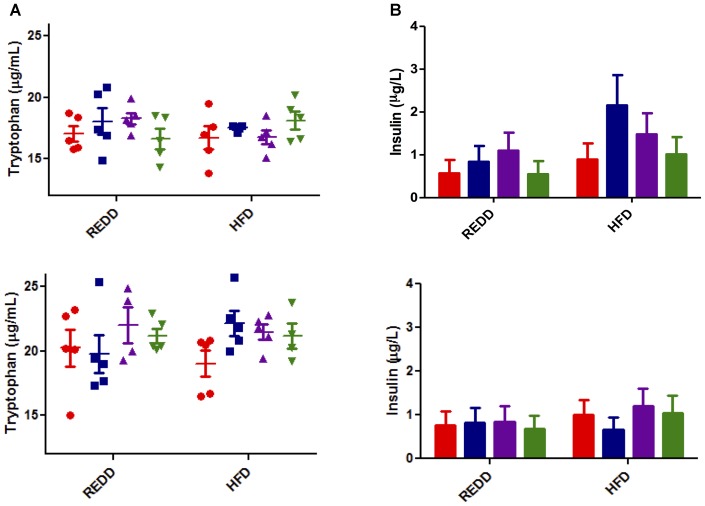
Analysis of mTORC1 inducers. **(A)** Tryptophan was quantified by LC-HRMS/HRMS from serum for male (top) and female (bottom). Red circle, control; blue square, blueberry extract; purple triangle, *L. johnsonii*; green triangle, blueberry extract + *L. johnsonii*. **(B)** Serum insulin was quantified by ELISA according to the manufacturers instructions for male (top) and female (bottom). For all insulin groups, *n* = 5. Red bar, control; blue square, blueberry extract; purple bar, *L. johnsonii*; green bar, blueberry extract + *L. johnsonii*. Numerical data are summarized as means ± SEM. REDD, reduced energy density diet; HFD, high fat diet.

To determine if the differences in mTORC1 expression levels were due to changes in external stimulatory factors or if they are intrinsic to treatments, common inducers to mTORC1 associated with diet and MetS were evaluated. Among these inducers of mTORC1 activity are insulin and insulin-like growth factor, inflammatory cytokines such as TNF-α, and amino acids, especially leucine and arginine (Ban et al., 2004; Lee et al., 2007; Vander Haar et al., 2007). However, a recent study has elucidated that tryptophan can act as a stimulatory signal for mTOR (Metz et al., 2012). Characterization of *L. johnsonii* N6.2 revealed that it produces a significant amount of hydrogen peroxide, which can modulate the activity of the rate-limiting enzyme in the tryptophan catabolism pathway, indolamine 2,3-dioxygenase (Valladares et al., 2013). Therefore, systemic tryptophan levels were evaluated to determine whether this was affecting mTORC1 activity. However, it was found that the systemic tryptophan levels did not significantly differ among treatments and diets, regardless of sex (Figure 7A).

Next, the levels of IL1β, TNF-α, and IL6 were determined in different metabolically susceptible organs. The induction of inflammatory cytokines IL1β, TNF-α, and IL6 are most notably associated with HFD-induced inflammation (Cano et al., 2009). Upon gene expression analysis of these genes in stomach, liver, and mesenteric lymph node tissue, these genes did not reach detectable levels sufficient to determine expression. Even though HFD feeding is associated with an increase in inflammation, it is possible that, as the rats were still in a pre- disease or -MetS state, inflammation is not evident.

Most associated with a HFD and MetS is an increase in circulating insulin as fats can induce glucose-stimulated release of insulin (Dobbins et al., 2002). Insulin is also a stimulator of mTORC1 activity. Analysis of circulating insulin levels indicated no significant differences among treatment groups or diet (Figure 7B). HFD moderately increased insulin levels in females, and treatment trends seem to be reflected between diets. Insulin levels in males seem to reflect relatively the same levels between the same treatment groups, excluding the spike in HFD treated with blueberry extract. Without the appearance of any significant trend, insulin levels do not seem to explain differences in mTORC1 activation. Therefore, it appears that the signal explaining the differences in diet and treatment groups still remains to be exposed, and could be more inherently associated with treatment qualities. Since treatments were not able to modulate external stimuli commonly associated with diet and MetS, it is possible that the mechanism of action of the treatments is their ability to directly interfere with mTORC1 pathway biomarkers. This, however, still remains to be elucidated.

## Discussion

The separation of animals based on sex revealed a perplexing realization: males were more susceptible to HFD effects, as is evident from significant increases in weight gain, blood glucose levels, and blood triglyceride levels, compared to female rats. Throughout the course of the study, HFD-fed female rats did not experience any significant differences in any of the MetS markers (Figure 2). It has been reported that estrogens, female sex hormones, can be protective against insulin resistance and glucose intolerance induced by a HFD, and that a decrease in estrogen is associated with an increase in abdominal fat (Riant et al., 2009; Sasayama et al., 2017). It is clear that the effects of the formulated diets are sex-specific, however, more investigation into this area is needed to see if estrogens are the sex-dependent culprits.

The difference treatments have on HFD-induced mTORC1 expression between males and females may be explained by one of two ways: (1) the differences may be a result of sex differences, or (2) the differences may be the result of MetS progression. In the later case, MetS progression is still rooted in sex differences, as males were more susceptible to a HFD. As mentioned, hormones are able to increase the expression mTORC1, one such hormone being insulin-like growth factor (IGF). It has been found that androgens are able to regulate IGF-1 transcripts in muscles of adult mice (Chambon et al., 2010). Furthermore, testosterone has been found to induce the IGF-1/AKT pathway by increasing expression of IGF-1 mRNA and glycogen synthase kinase 3β phosphorylation (Yin et al., 2009). Therefore, it is possible that the male associated sex hormones are causing the sustained increase in mTORC1 activation in males. In this study, the treatment of rats with blueberry extract and/or *L. johnsonii* was not sufficient to reduce mTOR activating phosphorylations in males as it was in females. The increased presence of androgens in males compared to females could be what is preventing treatments from having any noticeable effect in males.

An alternative explanation for the fact that treatments were unsuccessful in reducing HFD-induced mTORC1 activation in males is the possibility that males were at a risk of developing MetS, while females were not. Effects of MetS, such as increased insulin levels and lipid biosynthesis, could continually stimulate mTORC1. This could create such a high threshold of activation that the treatments of blueberry extract and/or *L. johnsonii* were not able to resolve this increased expression once it is triggered. This hypothesis is supported by the following observations: (1) treatments of blueberry extract and *L. johnsonii* were successful in reducing mTORC1-activating phosphorylations in females, while the combined treatment had a significant effect in reducing these phosphorylations (Figure 5), and (2) male rats that were fed a REDD showed decreased expression of mTORC1-activated genes when administered *L. johnsonii* and the combined treatment (Figure 6). Considering that a HFD did not increase MetS markers in females and assuming REDD-fed males were not at risk for developing MetS, these results may suggest that the diagnosis of MetS may influence the ability of treatments to reduce mTORC1 activation. Therefore, once the development of MetS is triggered, it could be more difficult to reverse the effects using only probiotics or phytophenol supplements.

A liver biopsy is still considered the gold standard in diagnosing NAFLD (Yoneda et al., 2017). Since we observed in this study that HFD-fed animals experienced increased lipid accumulation in the liver, it can be concluded that these animals have entered the beginning stages of steatosis. However, the progression from simple steatosis to NASH can be characterized by increased inflammation and oxidative stress in the liver. Sometimes coined as the “two-hit model,” simple steatosis could provide the background for the progression to NASH in some individuals, however, a “second hit” is needed to cause cellular injury and recruit inflammatory cells (Day and James, 1998). In this study, steatosis progression into NASH was evaluated by analyzing the inflammatory marker TNF-α, however TNF-α did not reach detectable levels. Some previous studies suggest the increase in TNF-α and the decrease of adiponectin, may be indicative of disease progression to NASH (Polyzos et al., 2016; Mohamed et al., 2017). However, no significant differences were detected between diet groups among males and females, indicating that animals are still in early stages of disease. Furthermore, a decrease in adiponectin levels upon HFD feeding has also been correlated with metabolic abnormalities, including insulin resistance in most animal models (Maeda et al., 2002; Nawrocki et al., 2006). This further reinforces the state of early disease onset in these animal models. However, some studies have presented normal insulin sensitivity in mice lacking adiponectin (Ma et al., 2002), so another marker in insulin homeostasis was assessed, *ceacam1*. HFD feedings have been shown in previous studies to reduce CEACAM1 mRNA levels by more than 50% after 21 days (Al-Share et al., 2015). This decrease was associated with hyperinsulinemia, insulin resistance, and elevated hepatic triacylglycerol content. Here, males, but not females, were observed to experience a more than 50% reduction in *ceacam1* levels, suggesting that, although in the early stages, males are more progressed into disease onset than females. Pre-disease onset is a critical period in a patient’s health, as intervention at this stage can prevent further disease progression. Dysbiosis has been associated with glucose intolerance (Suez et al., 2014), obesity (Le Chatelier et al., 2013), type 2 diabetes and insulin resistance (Qin et al., 2012), and alcoholic liver disease (Yan et al., 2011). Several bacterial feeding assays have been shown to reduce metabolic disease onset and its associated symptoms, such as obesity and glucose intolerance (Valladares et al., 2010; Vrieze et al., 2012; Rajkumar et al., 2014; Bagarolli et al., 2017). Since bacteria are able to modulate metabolic health, bacterial intervention during pre-disease onset could aid in preventing further disease progression during a pivotal time in a patient’s health.

Sexual differences played an important factor in the responses in this study. While diet is the significant factor influencing male responses, treatments are more influential in female animals. When analyzing males and females together, significant differences were found between the sexes in HFD treated animals for pAKT-T308 and pAKT-S473. Simply, males experienced higher mTORC1 activation in HFD-fed groups than females. As mTORC1 activation has been implicated in metabolic diseases, this increase could put males at a higher risk for developing health complications. However, comparing males and females on a REDD indicated no significant differences between treatment groups for these markers. Females on a REDD are arguably the group least expected to have metabolic complications in this study, since they appear to be more resistant to a HFD. As such, treatments’ ability to reduce these phosphorylations in males down to REDD females levels could indicate a protective effect in these groups. Downstream analysis of mTORC1-activated genes also reveals significant differences between sexes (*pgd, p* = 0.0023; *pfk, p* = 0.023). This data strongly suggests that effectiveness of treatments in modulating mTORC1 activation may be dependent on the sex of an individual. This information is critical when considering a study’s objective. A common practice in research is the use of only one sex in an animal study, then generalizing the findings to both sexes. However, it is impossible to predict if males and females will respond in the same fashion, because fundamentally, they are different. In the studies in which both sexes are used, their results are usually reported together, which has the potential to create bias if one sex has a particularly strong response compared to the other, or if a different number of each sex are used. This outcome can skew the data and lead to an incorrect interpretation of the results. This study highlights the importance of sexual differences, and provides evidence to be cautious when choosing animal models and deciding if it aligns with the study’s objective.

It is noted in this study that the HFD utilized was also effective in promoting mTORC1-activating phosphorylations in liver and pancreas. As these tissues are the center for regulating glucose and fatty acid metabolism, it is not surprising that a pathway regulating glucose homeostasis and lipid biosynthesis would be especially active in these tissues. Indeed, much research has evaluated mTOR pathway expression levels in these tissues under pathological conditions (Laplante and Sabatini, 2012). Future work will be focused on whether the same pattern of expression discovered in this study is reflected in these tissues as well. However, in this study we focused on our attention on the gastrointestinal tract, more specifically the stomach. The stomach provides an interesting and exclusive environment for our study. First, out of the entirety of the gastrointestinal tract, the stomach is one of the organs containing the fewest microbes. The richness of bacterial species in the rat stomach is lower than in any other organ of the gastrointestinal tract (Li et al., 2017). This can be attributed to the relatively low pH and the fast flow rate of contents through the stomach (McConnell et al., 2008). Among the bacteria that are able to persist in these harsh conditions is *L. johnsonii* (Tannock et al., 2012; Li et al., 2017). With the relatively low abundance of microbes in the stomach, the effects of *L. johnsonii* feedings will be amplified in this organ. We have also determined that the cinnamoyl esterases encoded by *L. johnsonii* remain functional in the emulsifying conditions of the gastrointestinal tract, and that the rat stomach is the first organ in which these released phenolic compounds can be absorbed (Lafay et al., 2006; Kin et al., 2009). These enzymes are still active in hydrolyzing fibers and releasing bioactive phenols from blueberry extracts. Furthermore, stomach tissue is among the first tissues *L. johnsonii* and released blueberry phenols come in contact with after ingestion. Therefore, the biological relevance of mTORC1 expression in the stomach and the effects of treatments in this organ are appropriate.

Bacterial supplementation has also been found to interfere with mTOR-related processes and genes. In a study analyzing growth performance, fat deposition, and lipid metabolism in *L. johnsonii*-fed broilers, it was found that bacterial feedings decreased serum triglyceride and abdominal fat, as well as reduced stearoyl-CoA desaturase-1, SREBP1c, and fatty acid synthase mRNA levels (Wang et al., 2017). mTORC1 promotes *de novo* lipogenesis through the activation of the transcription factor SREBP1c (Düvel et al., 2010) and can be linked to triglyceride synthesis through the effects of lipin1 (Peterson et al., 2011). Lipin1 can also induce the expression of many genes involved in adipogenesis (Koh et al., 2008). Stearoyl-CoA desaturase-1, which catalyzes the synthesis of monounsaturated fatty acids, and fatty acid synthase, which catalyzes the formation of long-chain fatty acids, are known target genes for SREBP1c (Peng et al., 2002; Mauvoisin et al., 2007). Since these genes and processes described in this broiler study are all tied to mTORC1 activity, a direct connection to *L. johnsonii* feeding and mTORC1 activity may be discovered in the near future. Another recent study on *Salmonella* Typhimurium, an intracellular pathogen, found that the depletion of energy during its pathogenesis triggers AMPK and other proteins needed to block mTOR and initiate autophagy to lysosomal degradation, thereby allowing *S*. Typhimurium to persist in the cell (Ganesan et al., 2017). This highlights the ability of bacteria to alter major regulatory pathways in the host for their benefit.

In this study, treatments of *L. johnsonii* and blueberry phytophenols were the most effective in reducing phosphorylations on AKT. Phosphorylation at AKT at Ser473 is required for full AKT activation and may facilitate phosphorylation of Thr308 by PDK1 (Sarbassov et al., 2005; Jacinto et al., 2006). mTORC2 is responsible for the phosphorylation of AKT at Ser473, and this phosphorylation unlocks AKT functions associated with glucose metabolism. AKT phosphorylation at Ser473 blocks the function of the transcription factors FoxO1/3a, which regulates cell proliferation, glucose metabolism, stress resistance, and apoptosis. The upregulation of FoxO1 in the liver has been associated with NASH, obesity, and insulin resistance, meanwhile hepatic FoxO1/3 knockout mice exhibit lower blood glucose levels and increased glucose tolerance (Rametta et al., 2013; Xiong et al., 2013). Given its association with MetS processes, therapeutic strategies have also been proposed to treat type 2 diabetes through FoxO1/3a signaling (Samuel et al., 2006; Nagashima et al., 2010). Although the focus in this study is monitoring metabolic disease progression through mTORC1, this study provides insight on potential regulation of FoxO1/3a, important metabolic factors, through the phosphorylation of AKT at Ser473 by mTORC2.

Since mTOR is a major regulator of a diverse set of cellular functions, and senses a diverse set of stimulatory signals, it is a challenge to isolate the signal leading to increased mTORC1 activation in a HFD context. Although we analyzed stimuli known to be associated with HFD-feeding and MetS (Figure 7), it is possible that a less obvious stimulus is causing these differences in activation. Certainly, there is much evidence in this study that could point to a sex-dependent source of difference, which needs to be further studied. If a difference in sex is the culprit, then this study further highlights the disparity between sexes in disease onset and provides insight on a possible mechanism from which this disparity stems. However, the differences observed could be a blend of sex differences and treatment effects. It is possible that *L. johnsonii*’s ability to release phytophenols from blueberry extract could be directly inhibiting key mTOR components, instead of an external stimulus. Many inhibitors of the mTOR pathway have phenolic scaffolds. For instance, LY294002 is a strong inhibitor of PI3K and is derived from the flavonoid quercetin, which has also been described to inhibit mTOR activity in cancer cells (Bruning, 2013). Quercetin is an abundant micronutrient in human diet, primarily found in fruits and vegetables, whose intake has protected animal models from HFD-induced weight gain and adipose tissue accumulation (Rivera et al., 2008). Quercetin and its derivatives make up a significant proportion of blueberry polyphenols (Borges et al., 2010). Additionally, proanthocyanidins have been shown to directly decrease AKT and S6K phosphorylations in esophageal adenocarcinoma cell lines and *in vivo* (Kresty et al., 2015). Many phytophenols have been the focus of recent research for their anti-proliferative properties in both cancer and MetS markers (Haghi et al., 2017; Zhao et al., 2017).

Overall, this study highlights the influence of sex as a critical variable in pre-disease onset. This study provides parameters that may help in the discrimination of responders versus non-responders usable in a future clinical trial. This study also highlights the potential use of a probiotic and a food supplement for the prevention of disease onset rather than for disease progression.

## Author Contributions

DK, LT, GL, and CG designed the research. DK and ED-S performed the research. DK, ED-S, SG, CG analyzed the data. SG provided the statistical support. DK and CG wrote the manuscript. All authors edited the manuscript.

## Conflict of Interest Statement

The authors declare that the research was conducted in the absence of any commercial or financial relationships that could be construed as a potential conflict of interest.

## Abbreviations

BBE: blueberry extract
HFD: high fat diet
Ljo: *Lactobacillus johnsonii* N6.2
MetS: metabolic syndrome
mTOR: mechanistic target of rapamycin
mTORC1: mechanistic target of rapamycin complex 1
mTORC2: mechanistic target of rapamycin complex 2
NAFLD: nonalcoholic fatty liver disease
NASH: nonalcoholic steatohepatitis
REDD: reduced energy density diet
S6K: p70S6 kinase 1
