# Supplementary material for: Sex Modulates Lactobacillus johnsonii N6.2 and Phytophenol Effectiveness in Reducing High Fat Diet Induced mTOR Activation in Sprague-Dawley Rats
Source: Front Microbiol. 2018 Nov 6;9:2649. doi: 10.3389/fmicb.2018.02649 (PMC6232610; doi:10.3389/fmicb.2018.02649)
Supplement: Supplementary file 1 [file Data_Sheet_1.DOCX]

Supplementary Material

Sex modulates *Lactobacillus johnsonii* N6.2 and phytophenol effectiveness in reducing high fat diet induced mTOR activation in Sprague-Dawley rats

Danielle N. Kling, Evon M. DeBose-Scarlett, Leandro D. Teixeira, Salvador A. Gezan, Graciela L. Lorca, Claudio F. Gonzalez^*^

*** Correspondence:** Claudio F. Gonzalez: cfgonzalez@ufl.edu


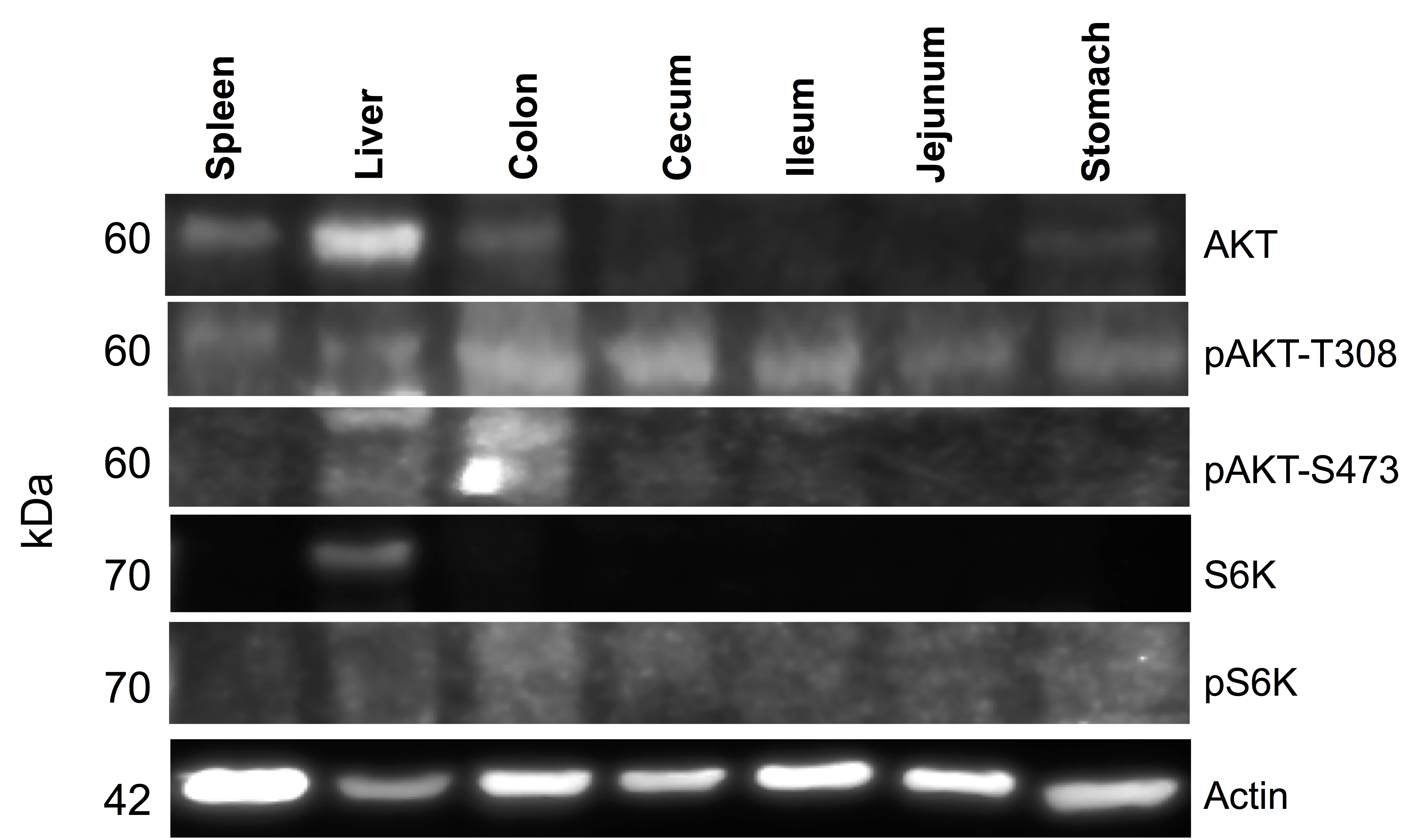


**Supplementary Figure 1.** Representative Western blot of the pattern observed in reduced energy density diet males comparing the expression of the mTORC1 pathway in the stomach, jejunum, ileum, cecum, colon, liver, and spleen organs. Actin was used as an internal control. All samples were run in parallel. Images were minimally adjusted in contrast and brightness.


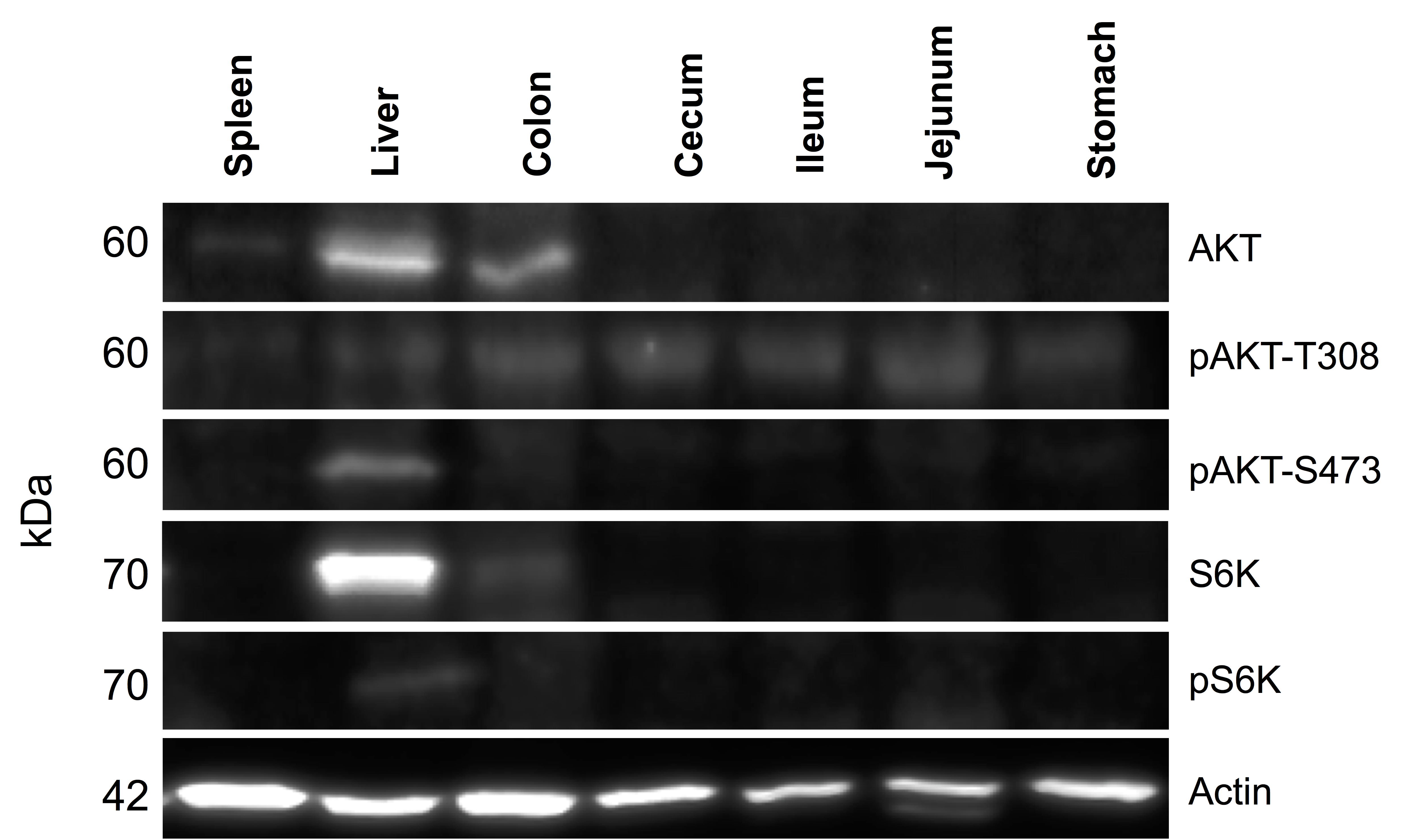


**Supplementary Figure 2.** Representative Western blot of the pattern observed in high fat diet males comparing the expression of the mTORC1 pathway in the stomach, jejunum, ileum, cecum, colon, liver, and spleen organs. Actin was used as an internal control. All samples were run in parallel. Images were minimally adjusted in contrast and brightness.


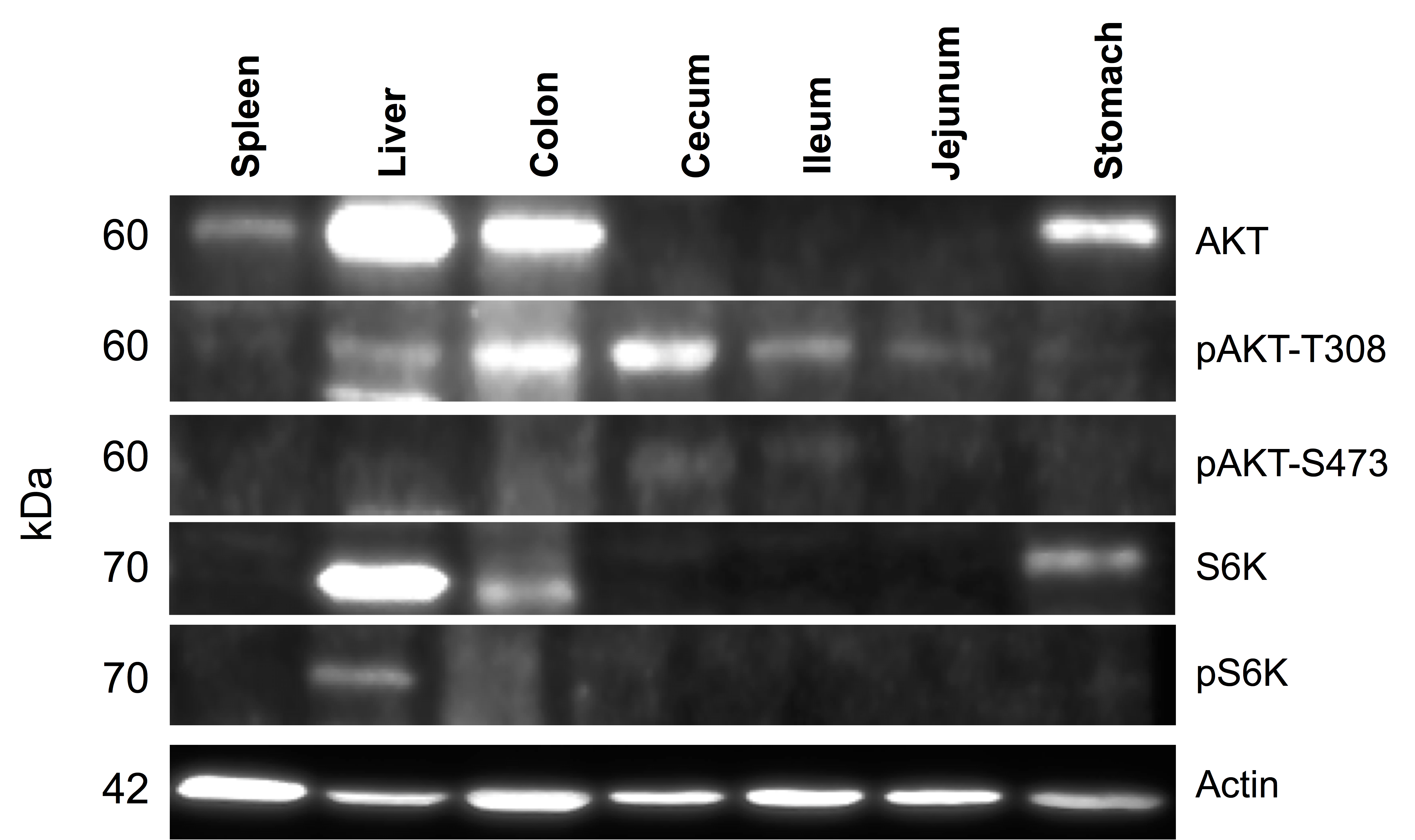


**Supplementary Figure 3.** Representative Western blot of the pattern observed in reduced energy density diet females comparing the expression of the mTORC1 pathway in the stomach, jejunum, ileum, cecum, colon, liver, and spleen organs. Actin was used as an internal control. All samples were run in parallel. Images were minimally adjusted in contrast and brightness.


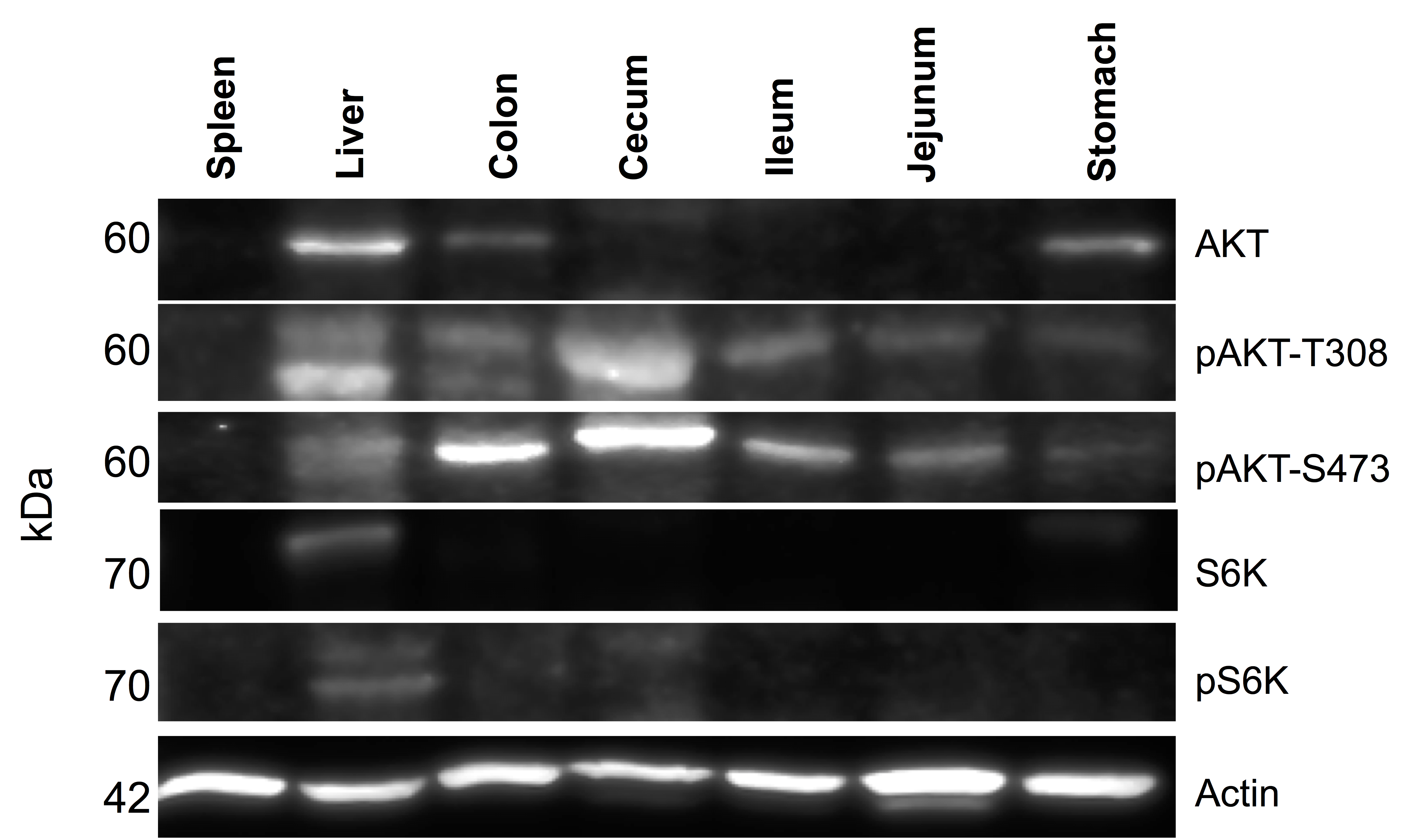


**Supplementary Figure 4.** Representative Western blot of the pattern observed in high fat diet females comparing the expression of the mTORC1 pathway in the stomach, jejunum, ileum, cecum, colon, liver, and spleen organs. Actin was used as an internal control. All samples were run in parallel. Images were minimally adjusted in contrast and brightness.


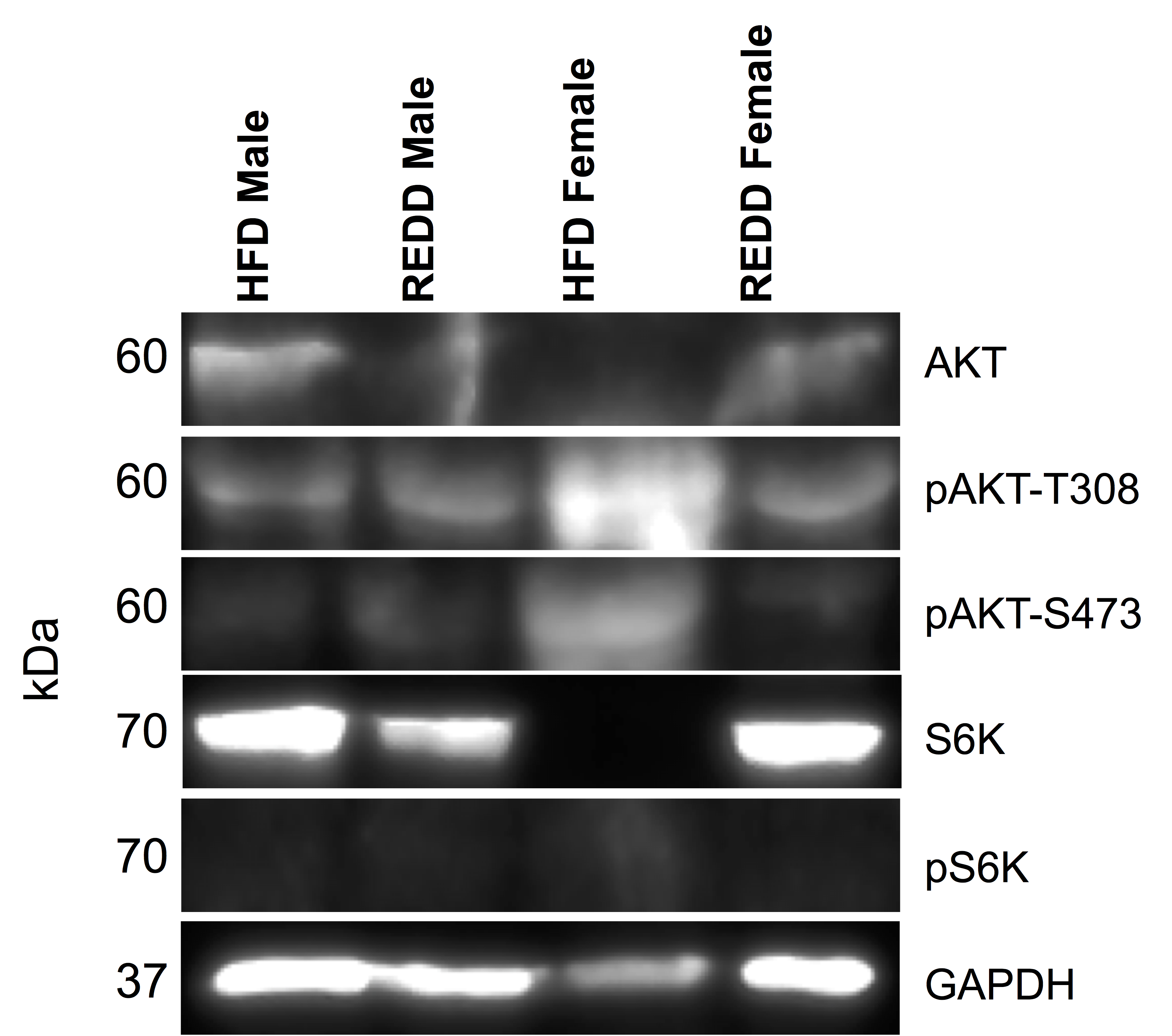


**Supplementary Figure 5.** Representative Western blot of the pattern observed in reduced energy density diet and high fat diet males and females comparing the expression of the mTORC1 pathway in the pancreas. GAPDH was used as an internal control. All samples were run in parallel. Images were minimally adjusted in contrast and brightness.
